# Counseling parents about child feeding: a qualitative evaluation of French doctors and health/childcare professionals’ experiences and perception of a brochure containing new recommendations

**DOI:** 10.1186/s12889-022-14778-2

**Published:** 2022-12-08

**Authors:** Sofia De Rosso, Camille Riera-Navarro, Pauline Ducrot, Camille Schwartz, Sophie Nicklaus

**Affiliations:** 1 Centre des Sciences du Goût et de l’Alimentation, CNRS, INRAE, Institut Agro, Université Bourgogne Franche-Comté, F-21000 Dijon, France; 2 Santé publique France, French national public health agency, F-94415 Saint- Maurice, France

**Keywords:** Feeding recommendations, Guidelines, Health communication, Health promotion, Complementary feeding, Qualitative research, Parental feeding practices, Breastfeeding, Public health, Pediatricians

## Abstract

**Background:**

Parents are crucial in establishing their children’s eating habits, and doctors and health/childcare professionals (HCCPs) can provide meaningful and trusted guidance on feeding, especially in the 0-3-year-old period. With the upcoming release of the official brochure containing the new child feeding recommendations in France, this study aims to: (1) assess professionals’ practices and perceptions regarding their communication with parents on child feeding and (2) evaluate their perception of the draft of the new brochure.

**Methods:**

A 15-page draft brochure (without pictures) containing updated child feeding recommendations for children 0–3 years old was developed by Santé publique France (the French public health agency). Online semi-structured interviews were conducted with professionals (*n* = 21), including 13 pediatricians and general practitioners (doctors) and eight healthcare or childcare professionals (HCCPs) two weeks after they were provided with this draft brochure to read. The interview guide was developed and piloted with other professionals (*n* = 3) prior to these interviews. Interview data were transcribed verbatim and analyzed thematically using an inductive approach.

**Results:**

While doctors and HCCPs mostly communicate orally with parents, both acknowledged that the brochure might be a helpful supplement, especially for HCCPs to legitimize their advice to parents. For doctors, giving the brochure to parents may help provide systematic advice and save time during consultations. Professionals serving parents of lower socioeconomic status would prefer a supplement with less text and more illustrations. In general, the messages were perceived to be easily understandable but providing detachable cards to distribute according to the child’s age would facilitate information dissemination and might be more useful to parents. Professionals reported that lack of training, the circulation of contradictory information, and language barriers were common challenges.

**Conclusion:**

French professionals welcomed the new official brochure as a means to spread updated child feeding recommendations. However, this brochure could be modified and specific tools developed to better adapt to professionals’ needs of communication with parents and to facilitate the relay of information. Providing updated and consistent information to parents should be considered a priority for public health stakeholders toward increased adherence to new recommendations.

**Supplementary Information:**

The online version contains supplementary material available at 10.1186/s12889-022-14778-2.

## Background

The first thousand days of life from conception constitute a crucial period in child development; this stage is characterized by rapid growth both in terms of physical evolution and behavior establishment [1]. Interventions targeting early childhood provide a unique window of opportunity to establish optimal infant nutrition and healthy eating behaviors [2, 3]. Moreover, promoting healthy lifestyles from infancy is paramount for the prevention of chronic conditions such as childhood obesity, of which the increasing prevalence in developed and developing countries is growing into a significant global health concern [4]. Childhood obesity, in addition to being a strong risk factor for adult overweight or obesity [5], is associated with an increased risk of comorbidities throughout life, including the development of diabetes, certain forms of cancer, premature all-cause and cause-specific mortality, and cardiovascular diseases [6–8].

Diet and physical activity are two factors directly associated with the development of overweight and obesity [9, 10]. These factors are influenced by the environment in which the child develops, as shown by Bronfenbrenner’s ecological systems theory [11]. Parents play a decisive role in shaping the eating behaviors of their offspring, among other behaviors [12]. For example, parents can modify children’s appetite self-regulation capacities [13–15]. Lack of responsiveness in parent-child interaction during meal times could impair the ability of the child to adjust his or her energy intake, often resulting in accelerated weight gain [16–18]. Parental feeding practices related to introducing vegetable are also likely to influence how much children like these foods [19]. Addressing lifestyle behaviors and feeding practices with parents early in children’s lives could help in the prevention of chronic diseases such as obesity and its health-related consequences [20, 21]. Public policies should account for the latest scientific contributions when addressing public health initiatives aimed at improving nutrition and health literacy toward evidence-based best practices [22, 23].

The need for guidance might alter parents’ ability to look for evidence-based information [24]. Parents seek information about child feeding from many different sources, including the internet, media, books and magazines, family, friends, doctors and health and childcare professionals [25, 26]. The type of information conveyed by these different sources can vary widely, and can even be contradictory [27], resulting in the spread of disinformation and misinformation. Considering that early eating habits generally have a lasting impact throughout childhood and continue into adulthood [28], it is essential to support parents with the appropriate tools and guide them to promote the healthy development of children. In France, official recommendations for feeding children aged zero to three were updated and published in 2019 in a format not suitable for the public [29, 30]. A paper brochure intended for parents and professionals was produced by Santé publique France (the French public health agency) to make these recommendations as accessible as possible to parents. A systematic evaluation of public health communication tools (such as this brochure) at the design stage is useful to account for the needs of recipients. Here, these recipients are parents and professionals. Professionals are judged by parents as a natural intermediary for the circulation of such information [31].

In France, when the child is between zero and sixteen years old, parents are encouraged to take her or him to 20 free and compulsory consultations with doctors, and the majority of visits take place within the first three years of life [32]. Moreover, other health and childcare professionals (HCCPs) play an important role in France for the support of harmonious child development: they interact with parents during the different phases of child feeding, and they are well placed to positively influence parenting decisions. The majority of parents in France trust pediatricians; as the 2013 Nutri-Bébé study showed, 58% of mothers of children between 15 days and 35 months seek advice from pediatricians on the best way to feed their child [33]. Moreover, a recent study in France showed that doctors are the most used and trusted source of advice (primary source of information for 81% of parents), followed by HCCPs who are less used (by 30% of parents) but very influent [31]. For these reasons, it is relevant to address doctors and HCCPs’ perceptions of public health material to support parental feeding decisions.

Little research has been conducted on understanding professionals’ practices and needs and integrating those needs to design effective prevention material for child feeding. Thus, the main objectives of this study were: [1] to investigate, via semi-structured interviews, the practices and needs of doctors and HCCPs when giving guidance to parents on child feeding (zero to three years old) and [2] to explore their perception on the draft of the brochure produced by Santé publique France, together with their level of understanding and acceptance of the new recommendations.

## Methods

### A brochure to spread child-feeding recommendations to parents

The French national agency for Food, Environmental and Occupational Health Safety (ANSES) published in 2019 an update of official guidelines for feeding children aged zero to three years mostly aimed at national agencies [29]. The process was supervised by the Ministry of Health, and in October 2020, the High Council of Public Health released a report reflecting these benchmarks [30]. The main changes in the new recommendations (tested with this study) compared to the previous ones [34] are summarized in Table 1. Subsequently, in 2019–2020, the content for a paper brochure intended for parents was developed by Santé publique France. This agency organized a group of experts with the intent of designing material to make the new recommendations as accessible as possible to parents, who are the ultimate users of this communication strategy. A qualitative study among parents to evaluate the content of the draft brochure (text and graphical principle) was also performed. The process of reformulating the recommendations and designing the brochure spanned November 2019 to July 2021, with the document becoming available to the general public in September 2021.

**Table 1:** Previous (2004) vs. new (2021) recommendations on child feeding contained in the official public health material (brochure) released in France

Topic	Previous recommendation	New recommendation
Start of CoF	Possible after 4 months	Between 4 and 6 months
Introduction of all food types	After 6 months	Between 4 and 6 months
Introduction of different textures	After 6 months	Between 6 and 8 months
Introduction of pulses and whole grain starches	Between 15 and 18 months	Between 4 and 6 months
Introduction of allergens (including gluten)	After 12 months	Between 4 and 6 months
Alternance of follow-on milk and full fat cow milk	Between 1 and 3 years, but to prefer follow-on milk	After the child turns 1 year
Baby-led weaning	/	No recommendation but this topic is addressed
Non-recommended foods and beverages^*^	To limit, and not before 7 months	To limit, to be introduced as later as possible
Fat	Never before 6 months	From the start of CoF
Responsive feeding	Not really addressed, from 6/8 months trust baby’s appetite	Trust the baby's appetite at all the ages; examples of baby’s hunger and satiety cues

*CoF* Complementary feeding

^*^E.g.: plant-based alternatives to formula, sugar sweetened beverages and foods, coffee, tea

A draft version of the brochure, comprising the core text (Word document) without pictures or illustrations, was provided to the professionals interviewed in this study. The document contained 15 pages organized in eight chapters, plus a table summarizing the food group introduction based on the age of the child. The first three chapters addressed generic topics pertaining to the development of eating behaviors of children and good practices for parents (including responsive feeding, discovering food textures, screen exposure); the remaining five chapters covered the development of feeding practices based on the age of the child.

### Study design

Doctors and HCCP’s experiences counselling parents about child feeding were undertaken using a qualitative inductive approach [35]. Face-to-face, semi-structured interviews were considered the best methodology to address the aim of the study. This approach could allow us to address specific questions in line with the research aims, but it could still give the freedom to the participant to talk about his or her experiences [36]. Given the context of the COVID-19 pandemic, interviews were scheduled online and conducted via videoconference when possible. In this way, it was easier to access participants in distant and different geographical locations, which allowed us to interview professionals from all over France [37]. Written notes were taken after each interview to highlight the attitudes or reactions of participants if necessary. The consequences of the restrictions imposed by COVID-19 on professionals’ practices and their interactions with parents (doing medical appointments via video or phone calls) were also assessed but will not be discussed in the present article. The consolidated criteria for reporting qualitative research (COREQ) checklist was applied to the description of methods and results [38].

### Participant recruitment

We aimed to recruit a sample composed of two-thirds of doctors, including pediatricians, general practitioners (GPs), and physicians working in maternal and child protection centers (PMI: *protection maternelle infantile*), and one-third of HCCPs, including professionals working in childcare centers, childcare assistants, and nurses working in PMI centers. Professionals were eligible if they were older than 18 years of age, practicing in France, and regularly interacting with young children (and consequently their parents) as part of their professional practice. Interns or retired professionals were excluded. To recruit participants, a purposive sampling method was initially used to ensure sample variety diversification: gender and age, profession and duration of professional experience, whether the participant has children, working in rural, urban, or semi-urban areas, and poverty rate of the working area. The objective was not to reach any quotas but rather to obtain a sample that was as balanced and diverse as possible. This method of recruitment was based on calls or emails to doctors and HCCPs in departments that were pre-selected according to poverty and urbanization rates, [39, 40] to include participants who dealt with parents of different socioeconomic status. Participants were also recruited by means of Facebook groups dedicated to French doctors and HCCPs (Additional file 1).

Considering other qualitative studies interviewing healthcare professionals [41–43], 21 participants were considered sufficient to ensure a diverse sample to explore the objectives of this study. The recruitment process determined if further recruitment was needed to ensure representatives from different professional categories.

### Data collection

Data collection took place in April and May 2021. An online demographic questionnaire was developed, it also included an informed consent form. The link to complete the questionnaire was emailed to those professionals whom volunteered to participate after first being contacted by the researchers (CRN or SDR) with a brief explanation of the study. The eligibility questionnaire was sent to 118 professionals, of which 40 completed it (25 Doctors, 15 CCPs). Of these, two doctors were not further contacted (one was unreachable; one was still an intern). After an eligibility check, the first volunteer participants who corresponded to the required profiles were selected and included in the study (*n* = 21), and none of them refused to participate in the study. We selected the professionals to diversify their socio-demographic characteristics, in particular the professions and the department (rate of poverty/urbanization) and the years of experience. For the doctors, we had few volunteers so little selection to make. If there were several participants with the same characteristics, we selected those who were the first to volunteer. Once the inclusion phase was completed, individual, semi-structured interviews were then scheduled for a date and time that suited the participants. The brochure draft was emailed to participating professionals at least two weeks before the interview to allow time to read it.

### Interview guide and procedure

The interview guide was first developed based on data found in the literature [44] and then was tailored to the objectives of the study. This guide and the study protocol were checked by a reference group of public health stakeholders (three members of Santé publique France with backgrounds in nutrition and policy communication). The interview guide was pilot tested with three preliminary interviews involving one doctor and two HCCPs. A preliminary analysis of these interviews allowed modifications and refinements of the script and provided an estimate of the duration of interviews. The interview guide comprised three main topic sections: [1] perceptions and practices related to the communication needs of professionals about child feeding; [2] changes in communication with parents due to the COVID-19 health crisis; and [3] comprehension of a brochure intended for parents and acceptance of the new recommendations. For each section, there were several core questions and probes. The translated interview guide is presented as Additional file 2.

Semi-structured interviews took place in the professionals’ native language (French). Interviews lasted an average of 54 ± 9 min. They were performed by the second author (CRN), a 25-year-old female, for the purpose of her thesis for her Master in Physiological and Psychological Food Choice Determinants. The interviewer had experience in health services research and engaging with doctors as a result of her prior studies in medicine. The interviews were conducted either using a videoconference system (Skype for Business) or via telephone (two interviews). During the interviews, the participants were at their home (*n* = 18) or workplace (*n* = 3), and only the participant and the interviewer were present during the recording phase. At the beginning of each interview, CRN introduced herself (name and workplace) and gave a short introduction to the study and the data protection policy. The participants did not know anything else about the interviewer. Professionals had to verbally confirm that they agreed to be audio recorded. After being asked all the questions regarding the interview topics, professionals were invited to share any additional information that they considered important. When the participants had not read the brochure prior to the interview (*n* = 2), the interviewer gave them time to read it, either by postponing the interview or by calling for a break during the session, according to participant’s preference. When there was nothing to add, participants were thanked for their time, and the following steps of the study were explained. The interviewer took some notes after each interview and had regular discussions with SDR, the PhD student (31-year-old female) with a background in public health and nutrition who oversaw this study and who contributed to the conception of the brochure and participated in the previous phases of the brochure draft evaluation (e.g., focus groups with parents, evaluation of effect of the brochure on parental knowledge). All interviews were audio recorded and transcribed verbatim. After all the interviews concluded, the participants were sent a brief summary of the study results and were asked to refer to the researchers if they had anything to add or disprove. Participants did not provide additional feedback on the report to the researchers.

### Ethical consideration

All the participants received a written description of the project and were informed of their right to withdraw at any time without prejudice. Professionals agreed to participate by giving their informed consent and selecting the appropriate case in the online form. To ensure confidentiality of the information collected and anonymity, the participants were assigned a code that only the interviewer could match to their names. These codes were erased once the interviews were completed, and only sociodemographic characteristics were retained. Each participant received a voucher worth 40 Euros as a token of appreciation. Ethical approval (n°21–788) for this study was obtained from the Institutional Review Board (IRB00003888, IORG0003254, FWA00005831) of the French Institute of Medical Research and Health. This study was registered by the data protection service involved.

### Data analysis

The audio records of the interviews were transcribed verbatim by a professional transcription service (Le Scribe Audio, 11/21 interviews) or by the interviewer (CRN) with the help of a research technician with experience in transcribing interviews. All verbatim data were double checked for accuracy by the interviewer and the first author (CRN and SDR). The software NVivo was used to analyze the interviews in their original language to avoid loss of meaning. A thematic analysis was conducted in accordance with the six steps proposed by Braun and Clarke [45]: (1) data familiarization, (2) initial coding generation using a data-led approach, (3) searching for themes based on initial coding, (4) review of the themes, (5) themes final definition and labeling, and (6) report writing. The familiarization step was performed throughout the data collection phase by the first two authors (SDR, CRN), who both listened to recorded interviews and read the transcripts. The first author discussed with the interviewer on a regular basis during the interview phase to brainstorm about the themes based on emerging patterns (main ideas) in the data. The first three steps were performed manually and independently by SDR and CRN. Initially, eight interviews were selected for independent initial coding (5 doctors, 3 HCCPs). Coding was performed with a data-led approach, with particular attention to include the topics addressed in the interview guide. At the completion of this coding phase, the emerging themes and subthemes were discussed with the other authors until a consensus was reached and a coding scheme was created. The coding scheme was used to complete the coding of all the other interviews, which were coded independently by SDR and CRN. When all interviews have been coded, if new codes emerged then the final coding scheme was also used to adjust the coding of the first eight interviews. SDR and CRN subsequently compared the coding of all the interviews and evaluated whether there were doubts or difficulties regarding the placement of quotes. Then, a discussion took place until an agreement was reached, and all the codes were homogenized. At the end of this process, SDR and CRN once again reviewed the themes and subthemes and defined those that were relevant for answering the research questions of the current study. A final decision was made after discussion with SN. Data were analyzed in two parts: (1) discourse dealing with doctors and HCCPs practices and needs in terms of communication with parents about child feeding and (2) discourse dealing with their perception of the brochure and its evaluation.

## Results

Table 2 summarizes the characteristics of the 21 participants. Interviews were undertaken with a total of 13 doctors (nine women) and eight HCCPs (seven women) from a range of disciplines.

**Table 2:** Characteristics of the sample of French professionals (*n* = 21)

Characteristics		All (n)	Doctors (n)	HCCPs^1^ (n)
**Totals**		**21**	**13**	**8**
*Profession*	Pediatrician	5	5	**/**
	General practitioner	5	5	**/**
	PMI physician	3	3	**/**
	Childcare assistant	2	/	2
	Professionals working in childcare centers	3	/	3
	Childminder	1	/	1
	PMI nurses	2	/	2
*Gender*	Female	16	9	7
	Male	5	4	1
*Age range*	Less than 35 years old	11	8	3
	35–49 years old	7	4	3
	More than 49 years old	3	1	2
*Years of professional experience*	0–10 years	14	11	3
	11–20 years	4	1	3
	21–30 years	2	1	1
	> 30 years	1	/	1
*Number of children*	0	6	5	1
	1	5	2	3
	2 or more	10	6	4
Poverty rate of the department (%) ^*^	< 13.3%	9	6	3
	13.3 -17.2%	8	5	3
	> 17.2%	4	2	2
*Urbanization rate of the department (%)*	40–80%	7	4	3
	> 80%	14	9	5

^1^ HCCPs = other healthcare and childcare professionals

^*^Poverty rate: the monetary poverty rate corresponds to the proportion of individuals (or households) in a given department in a situation of monetary poverty. An individual (or a household) is considered poor when he lives in a household with a standard of living below the poverty line. In France and in Europe, the threshold is most often set at 60% of the median standard of living

Two main themes and nine subthemes were developed through the thematic analysis. The main themes were: (1) practices and needs in terms of communication with parents on child feeding, and (2) perceptions of the brochure. Regarding practices and needs, four subthemes were identified: communication practices, communication barriers between professionals and parents, needs of professionals and needs of parents to improve communication. Regarding perceptions of the brochure draft, subthemes included new recommendations, general impressions, suggestions to improve the brochure, perceptions of the utility of the brochure in doctor and HCCP practices, and in parents’ practices. Detected differences between doctors and HCCPs’ discourse were reported in the results. The themes and subthemes are illustrated in Table 3 and described in detail below.

**Table 3:** Overview of themes and subthemes

Themes	Subthemes
1) Practices and needs in terms of communication with parents on child feeding	*Communication practices*
	*Communication barriers between professionals and parents*
	*Needs of professionals to improve communication with parents*
	*Needs of parents*
2) Perceptions of the draft brochure	*New recommendations*
	*General impressions of the brochure (positive and negative)*
	*Suggestions to improve the brochure*
	*Perceptions of the utility of the brochure in Doctors and CCPs practice*
	*Perceptions of the use that parents can have of the brochure*

### 1) Practices and needs in terms of communication with parents on child feeding

#### Communication practices

Most of the professionals mentioned that they mostly provided information on child feeding orally to parents. Several doctors (*n* = 8) also suggest websites to parents where they could search for information ( the most cited websites are listed in Table 4). All doctors explained that they systematically spoke with parents about child feeding at regular consultations within the first year of life, especially on complementary feeding (CoF) when the child is from four or five months of age. They mentioned that they do not have a rigid approach on how and when parents should start CoF. However, almost all doctors reported that they liked to provide parents with a framework (starting with fruits and vegetables and introducing proteins after six months of age).

**Table 4:** Websites cited by doctors (from top to bottom, the most to the least cited)

Websites
Mpedia.fr
Pediadoc.fr
MangerBouger.fr
Pédiatre-online.fr
Ameli.fr
Pap-pédiatrie.fr
Alimentationdutoutpetit.fr

*“So, I recommend starting at five months but only with vegetables, and then introducing fruits. After six months, introduce animal proteins such as meat, fish, and eggs.” (P2, Doctor, General practitioner)*.

Some doctors mentioned that exchanges about child feeding become less frequent and less systematic after one year. The media used by doctors to support oral communication are the child health record booklet, paper documents adapted to the child’s age range, and websites that parents can visit (*see* Table 4). Professionals working with low socioeconomic status families (especially in PMIs) explained using Google during consultations to show pictures on the computer screen and to translate their speech.

“We *adapt to families, for example when we recommend whole milk, we put pictures on the computer, we say “the red cap”, or “the pink cap” for follow-on milk; finally, we manage to find solutions.*” *(P20, HCCP, PMI nurse*).

Regarding HCCPs, very practical information on child feeding is exchanged with parents (what the child eats at home and at the childcare center). This information is given especially orally during the morning (when parents drop off their child) and evening “transmission” times (when parents pick up their child), but also with the support of paper documents.

#### Communication barriers between professionals and parents

Language was mentioned by some professionals (*n* = 9) as a barrier to communication with parents, especially in PMIs, where professionals meet families with the lowest socioeconomic status (often with a foreign origin).

*“One of the difficulties we have at the PMI is that we have a lot of very precarious families […] therefore they face many difficulties other than the problems linked to their daily diet.” (P12, Doctor, PMI physician).*

The lack of time and training (inconsistent training done on a voluntary basis) were also mentioned as barriers by some of the professionals (lack of time: n = 6 (all doctors); training n = 7).

*“It’s supposed to be our role, but we don’t necessarily have the training, support or the time.” (P7, Doctor, General practitioner)*.

It was also stated that the perpetual change in the child feeding recommendations confuses parents and impairs communication, since it induces the circulation of conflicting information, even among professionals. Socioeconomic insecurity was reported to impact communication on child feeding.

#### Needs of professionals to improve communication with parents

Some of the professionals mentioned that they needed “official documents” and nutrition training to improve their communication with parents to standardize the knowledge related to infant and young child feeding.

*“There are a lot of different opinions that are not necessarily very serious and not very trustable, but I think that by standardizing the opinions, we would have more credit and it would be easier for parents.” (P8, Doctor, General practitioner)*.

Professionals working in PMIs emphasized their need for more adapted media (in particular, a short document with many illustrations and few texts and/or in various languages).

*“In fact, we would need either adapted documents in several languages, or much simpler things. We really need things that are quick and simple to show to parents. And that’s kind of what we’re missing.” (P20, HCCP, PMI nurse)*.

#### Needs of parents

Several professionals reported that parents are asking for information on feeding, especially CoF. They noted that some parents need very specific guidance from doctors, while other parents prefer flexibility in the information they receive. Parents ask for details on the recommended quantities of food and milk as well as practical advice (e.g., how to introduce an allergen).

*“In fact, the majority really like flexibility, and there is a small minority who hate it, and who prefer to have very, very simple and very precise instructions.” (P1, Doctor, Pediatrician)*.

### 2) Perceptions of the brochure

#### New recommendations

All professionals were aware that it is now recommended to start CoF between four to six months of age to prevent allergies. However, when a mother was breastfeeding, professionals were more likely to recommend starting CoF at six months of age in accordance with the World Health Organization (WHO) recommendations (encouraging exclusive breastfeeding until six months of age).

*“Sometimes breastfeeding mothers want to continue exclusive breastfeeding up to six months. In general, I am not really against it because the WHO recommends that. So, I don’t have too many reasons to go against it especially if it’s desired on their part.” (P2, Doctor, Pediatrician)*.

Recommending introducing all types of food between four to six months of age was still not grounded in practice among the interviewed professionals. Professionals were used to advising introducing proteins from six months of age, as suggested in the previous recommendations. Some doubts about the early introduction of allergens and proteins were expressed.

*“Being able to introduce all the foods is the biggest novelty. We can introduce everything, taste more or less anything, in particular and at the same time, without respecting… waiting for the six months.” (P8, Doctor, General practitioner)*.

*“What’s the point of wanting to absolutely start protein right away between four and six months? […]. The info on the introduction of peanuts, which I used to do previously, but is it really scientifically validated? Because they don’t all have the same indications about it between allergy organizations.” (P5, Doctor, Pediatrician)*.

According to the new brochure, pulses and whole grain starches can be introduced to children from four months of age. This recommendation sounded relatively new to professionals, but it appeared to be well accepted despite some doubts about their digestibility were raised. Some HCCPs noticed that feeding practices in childcare centers do not meet this recommendation.

*“I discovered that at four months you could give that: lentils and mashed beans. I did not know. It was complicated for me because the fiber and the lentil, bean, or chickpea skins have to be very well mixed. But I find it good, it’s interesting.” (P21, HCCP, PMI nurse)*.

Most of the professionals knew that it was possible to alternate follow-on milk and full-fat cow milk after the child turned one years old. They found that this was a good option to propose to families with low socioeconomic status. However, there was a tendency to emphasize follow-on formula to avoid iron deficiency. The purpose of this recommendation seemed to be less understood by HCCPs.

*“And I had to do it before because I know that you can give whole cow’s milk instead of follow-on milk. […] On the other hand, it is true that where I insisted, it was that it should not be exclusively cow’s milk. They need a lot of iron and that is not covered by cow’s milk.” (P9, Doctor, General practitioner)*.

*“I think there is ambiguity, and we are not sure if we are completely marketing or if this follow-on milk really brings more to the children. And again, from one pediatrician to another, we can see that there are two schools, there are really those who switch to UHT milk without worry and others who are reluctant.” (P14, HCCP, Professional working in a childcare center).*

All professionals were familiar with non-recommended foods (e.g., plant-based alternatives to formula, sugar sweetened beverages and foods). Some of them asked for more explanation, especially regarding the new recommendation about avoiding chocolate until three years old.

Professionals mentioned that they particularly enjoyed one section of the brochure on baby-led weaning as they face an increasing number of questions about this topic and need more evidence-based information.

*“As I read, I also thought about the baby-led weaning, I wondered if it would be covered when I saw that there was a little passage on it. It’s true that sometimes it’s not very common, but it so happens that there are parents who say they want to do the baby-led weaning when in fact, frankly, I have no knowledge of it. I do not know what to advise them, as it is really outside the framework of the recommendations that I’ve read.” (P2, Doctor, Pediatrician)*.

*“Then, we have more and more questions about baby-led weaning. This is something I had never heard of… I have the feeling that a year ago we didn’t talk about it, and in the last year we have talked about it a lot. There is a little insert on it, that’s good.” (P17, HCCP, Childcare assistant)*.

#### General impressions of the draft brochure

Positive impressions of the brochure outweighed negative impressions (see Table 5). In fact, all 21 professionals expressed at least one positive impression, and overall, they seemed enthusiastic about the brochure (some of them wanted to use it forthwith).

**Table 5:** Summary of the general impressions about the draft brochure. Total of participants *n* = 21

Positive impressions	Negative impressions
Complete and precise document (*n* = 15)	Long document (parents might consequently not read it) (*n* = 12)
Positive tone (not judging/guilt-inducing) (*n* = 10)	Layout (participants were provided with a draft (only with text)) (*n* = 8)
Agree on the topics order (from general to age-adapted information) (*n* = 9)	Summary table is complex and difficult to read (*n* = 7)
Global approach including advice on screens, physical activity (etc.) and parenting advice (*n* = 9)	Some recommendations are still guilty-laden (recommendations on breastfeeding, organic foods and industrial foods) (*n* = 3)
Summary table is well done and useful (*n* = 7)	Unsuitable/Improper advice about child motor development (*n* = 3)

#### Perceptions of the utility of the brochure in doctor and HCCP practice

Doctors considered the brochure as a supplement that could facilitate communication with parents. Using the brochure could help save time during consultations (parents could read the brochure at home and return to the next consultation with additional and more precise questions). Moreover, the brochure could enable doctors to have a more systematic approach in their information delivery to parents about child feeding, and help doctors update their knowledge of the new recommendations.

*“I find that supports our speech by saying: “It’s not just my personal opinion and what I personally believe. This is really what is recommended”.” (P8, Doctor, General practitioner)*.

*“That [reading the brochure] clarifies the recommendations, so that’s great. I am very happy with it. […] I learned some things.” (P9, Doctor, General practitioner)*.

In PMIs, the brochure could be used only with a few parents because it is too long and complex for serving the families with lowest socioeconomic status. HCCPs generally stated that they would give the brochure to parents who have specific questions about infant and young child feeding. The brochure would help HCCPs feel more legitimate and precise; improving their skills in giving advice to parents.

*“These are somewhat formal documents; we still say to ourselves that it was thoughtfully studied by professionals. It gives us legitimacy and technical consistency.” (P14, HCCP, Professional working in a childcare center)*.

#### Suggestions to improve the brochure

Most of the participants recommended making the brochure more entertaining (with pictures and less text) as they were provided with a preliminary version of the brochure with no illustrations (Word document). Participants suggested translating the brochure in different languages or to make one document with visual explanations and many pictures adapted to PMI populations. Participants also proposed making different detachable sheets adapted for each age range of the child to easily distill the information to parents. Including the brochure in the child health record booklet and adding more explanations and/or scientific references (e.g., why starting CoF between four to six months of age help prevent the development of allergies; why is it recommended to avoid salt until three years of age; scientific references to justify the introduction of gluten and nuts, etc.) were also popular suggestions.

*“In fact, we should be able to cut down the number of items to give just the one that corresponds to the needs of the families in the moment. After that there is a need to put images so that we can adapt it to our families.” (P20, HCCP, PMI nurse)*.

#### Perceptions of the use that parents can have of the brochure

Professionals believe that the brochure is a comprehensive and useful written tool for parents and can serve as a reference for discussing child feeding with doctors. They believe that the brochure will be more useful for parents who are already invested in the topic of child feeding and that its usefulness will be more limited for parents with lowest socioeconomic status. The brochure could encourage parents to change certain habits (e.g., to cook more homemade dishes).

## Discussion

In this qualitative study, we interviewed French doctors and healthcare and childcare professionals who serve young children and their parents. The first aim was to explore their practices and needs when communicating with parents about child feeding from zero to three years of age. The second aim was to explore professionals’ impressions regarding a draft brochure containing the newly updated child feeding recommendations and focused on understanding acceptance of the new nutritional recommendations. We decided to focus specifically on doctors and HCCPs in recognition of their central role in guiding and influencing parents in the early life of children [31].

The results of this study suggest that overall this paper brochure meets professionals’ needs. Positive opinions outweigh negative ones, even if the brochure was described as too long by several professionals. The fact that it was a paper document was well appreciated by the majority of the professionals. This is in line with the results of a recent double survey regarding the perceived needs of French parents and pediatricians concerning information on CoF, in which 59% of pediatricians and 42% of parents considered paper brochures to be an appropriate tool to spread information regarding CoF [27]. Professionals indeed reported they would use the brochure as a medium to communicate with parents, except in serving PMI populations where a simpler and more colorful document was requested.

There were some differences in terms of communication practices between HCCPs and doctors, especially concerning the content of exchanges with parents. While HCCPs tend to discuss more practical topics such as daily issues related to the diet of the child, doctors tended to discuss more feeding-related topics. On average, pediatricians and GPs perceived the brochure as more useful in their practice than HCCPs. Those dissimilarities in practices between HCCPs and doctors and their perceptions of the utility of the brochure could reflect the different roles of employment between these two professional categories. Specifically, while doctors reported they could use the brochure in a systematic way with parents during consultations, HCCPs would rather use it as an instrument to inform themselves, legitimize their knowledge on the subject, or to assuage the doubts of curious parents, confirming HCCPs’ need for additional training and education. Nevertheless, both types of professionals agreed that this brochure would enable them to update their knowledge about nutrition for children from zero to three years of age.

In PMI centers, the story is completely different from that in pediatricians’ offices. In fact, the professionals dealing with low socioeconomic status families expressed strain in having to use the brochure with their patients. Often parents visiting PMI professionals do not fluently speak or understand French as they are immigrants or of foreign origin ; in those cases, an adapted tool with more pictures and images is required to facilitate communication. The lack of tools that account for the needs of those parents was previously noted in another study in which 35% of pediatricians thought that communication materials available to parents do not sufficiently consider the different economic and cultural situations of families [27]. In a similar way, Norwegian public health nurses identified language and cultural barriers as common challenges in counseling immigrant parents about food and feeding practices, suggesting that culturally adapted information materials translated into different languages and visual aids containing as little text as possible may facilitate communication. Based on these results, they developed an image-based communication tool for universal use. As reported in individual interviews of 12 non-immigrants parents, this tool showed benefits even with non-immigrant parents, to provide them with fundamental basic knowledge, such as informing of healthy food choice or to present applicable portion sizes for children. However, this study also highlighted that a two-way dialogue was also required in order to adapt nutrition counseling to the needs of each family and to encourage discussing topics such as feeding practices. It was already demonstrated that public health campaigns focused on diet and nutrition struggle to reach those of modest means [46, 47]. This aligns with data showing that chronic conditions such as obesity and diabetes are more prevalent within the lowest socioeconomic classes [48, 49]. The challenges of involving a low socioeconomic status population in public health campaigns could exacerbate social health inequalities [50]. To tackle this issue, the campaign regarding the new recommendations, which launched in September 2021, also included other mediums of communication, such as videos. These tools can overcome language barriers and may increase parents’ capabilities to turn recommendations into practice by providing concrete advice and examples for feeding children. Disseminating health messages through doctors and HCCPs was also a means to efficiently reach all parents (including those of low socioeconomic status). A previous study has indeed shown that doctors and HCCPs are the most influential sources of information to parents regardless of their financial status [31]. In addition, in the near future, other tools are planned to be designed to specifically address the needs of low socioeconomic status populations as identified by professionals. Moreover, in France, the program MALIN (www.programme-malin.com) is now disseminated through the family allowance with the objective of promoting healthier food practices for children under the age of three who come from families who meet financial difficulties [51]. Families with low socioeconomic status, who meet the eligibility criteria (e.g., income), could be provided vouchers to buy baby food or groceries and given advice, to empower them to make better nutrition-related choices for themselves and for their children.

This study did not reveal important differences between the new recommendations and the advising practices of professionals (especially doctors). However, it still pointed out particular advice given by professionals that could be updated and improved in light of the new recommendations. In particular, with regard to the introduction of allergens and of all types of food between four to six months of age. Our results are in line with those of a study conducted in France in 2016, in which the interviewed general practitioners and pediatricians reported giving advice on child feeding in line with recommendations [52]. For instance, in this study, professionals acknowledged that the right age for introducing complementary food was between four and six months of age and gave the appropriate advice to parents in 85.2% of cases. However, in the same study, the given advice was not in line with recommendations as was highlighted regarding the introduction of allergens and some foods. For example, 44.5% of professionals were advising parents to introduce gluten to their child after six months of age [52]. Additionally, in the case of parental allergy history, there was a tendency to tell parents to postpone the introduction of some specific foods. Some of the professionals interviewed in our study also experienced doubts regarding the introduction of allergens. An uncertain opinion from a professional could create confusion regarding the time of introduction of complementary foods and could introduce non-compliance in parents due to reasons such as lack of confidence in the doctor and preference to follow advice from family or friends [53, 54]. The uncertainty experienced by some professionals facing the newest recommendations underscores the time required for scientific, evidenced-based, knowledge to reach all the intended recipients. A consensus among professionals on child feeding recommendations could improve parental confidence to follow the given advice, but this remains a difficult objective to achieve. Santé publique France has been moving in this direction with a communication campaign targeted toward professionals with the goal of raising their awareness to the new recommendations prior to the launch of the campaign targeted toward parents (August 2021).

The brochure produced by Santé publique France is an official document that formalizes and standardizes the latest recommendations for feeding children from zero to three years of age. It is interesting to note that the brochure itself did not fulfill the doctors’ need for explaining some recommendations, since they expressed the need to verify some of the information themselves. Chouraqui et al. also reported the need of GPs and pediatricians to obtain more information about certain infant feeding practices, more generally on specific pathology-related recommendations (such as the prevention of allergies, the age of gluten introduction, and the treatment of food allergies) as well as weaning and the introduction of foods according to the child’s age [52]. Interestingly, in our study, GPs and pediatricians also reported their need to obtain more information regarding the quantities of milk according to the child’s age to answer parents’ questions, which suggested that this recommendation about milk quantities still creates confusion. Professionals also needed more explanations regarding why some foods are not recommended to young children (e.g., certain types of fish, chocolate). Banti et al. in 2015 surveyed French pediatricians to understand whether their practices followed official recommendations. It emerged that frequent changes in the recommendations could be perceived as errors [55]. This might reduce the level of trust in updated official documents, and consequently, the given advice may no longer align with the latest evidence. Clear explanations are requested to detail why recommendations had changed in comparison to the previous ones. In this case, scientific references are requested, especially by doctors.

Our results show that professionals believed that they have a role to play in the dissemination of the brochure. Indeed, the discourse of doctors suggests that the brochure could help overcome some of their communication barriers. In fact, providing the brochure to parents would allow doctors to save time during consultation (parents would have all the necessary information in the document so doctors focus on talking about the most important topics and save time to answer questions) and to overcome the lack of practical advice. In addition, using the brochure as a support to communicate with parents would help doctors and HCCPs provide more complete and systematic information about child feeding and bring more legitimacy to their communication with parents. In serving PMI populations, the main barrier to communication with parents was language. However, a written support with images and few words could facilitate communication with foreign or immigrant families. The development of other means of dissemination of information might be needed to foresee an inclusive strategy at every population level. The perspective of creating digital solutions has been discussed previously in the literature, but doubts still exist on how to deal with social disparities in terms of digital health [56].

Strengths and limitations must be analyzed when considering the results of our study. For the design of the study, we aimed to include unequal numbers in different professional categories. This decision was based on another recent study, as explained above, but this consideration should be taken when interpreting and generalizing the findings of our study. A potential bias could emerge when interpreting findings by giving undue prominence to views of certain professions (mostly GPs and pediatricians). Notwithstanding, during the analysis and presentation of the results, efforts were made to ensure that points at which views and perceptions differed across professional groups was explicitly stated. Additionally, as often happens with qualitative studies, social desirability bias could be a limitation; in fact, professionals might have had the instinct to say something just because it was perceived to be the “correct” answer according to their perception of their role. In addition, an effort was made to include a high number of doctors (GPs, pediatricians, PMI physicians) across a spread of different departments in France. In fact, this population is often difficult to involve in research studies due to work schedules that often cannot accommodate activities other than outpatient practice. Moreover, while we included a wide variety of professionals, other categories of doctors and HCCPs, such as midwives, could have been involved. Our inclusion method occurred mostly via social media, which yielded heterogeneity in terms of the age of the participants, which was not representative of the national population. Finally it worth noting that the draft version of the brochure that was being tested at the time of the study did not present any graphical adaptation yet. Although, visuals are often important components of education material, as indeed mentioned by many participants. In this project, the present study was one-step of many in the brochure evaluation process. During further steps, the brochure was evaluated in a fully designed form before being disseminated.

## Conclusion

Early life is an important period for the development of healthy behaviors in children, especially regarding eating. Involving health professionals in a structured strategy of disseminating child feeding recommendations can be important given the high frequency of routine contact that they have with parents in their child’s early years. This qualitative analysis conducted with health and childcare professionals allowed us to explore their practices and needs in terms of communication with parents on child feeding. Professionals reported that while communication with parents primarily occurs orally, they also often refer to other supports (e.g., pamphlets, online sources). Lack of time and language were major barriers, especially in PMI centers that are serving low socioeconomic status families. Moreover, this study investigated whether professionals found the draft brochure developed by Santé publique France suitable to meet their needs, whether they could easily integrate the new recommendations when advising parents, and whether they considered that additional adaptations are needed. Our results show that professionals generally welcomed the brochure, but this medium was still considered as a tool that was not well-suited for families with low socioeconomic status, for whom a different support that includes more visual representations should be developed. A strategy that entails the use of the same document by both parents and professionals has the advantage of harmonizing the information circulating between them. Professionals would feel more legitimate in advising parents with the help of a document enclosing recommendations supported by all the newest scientific evidence. Moreover, providing relevant and consistent information to parents could help reduce the flow of conflicting information and misinformation and ultimately increase parental support for adopting new recommendations in their child feeding practices. To reduce health inequalities, further actions are needed from the public health sector to more specifically address the low socioeconomic status population. In the long run, this may contribute to reducing social disparities in nutrition and health.

## Supplementary Information

**Additional file 1. **Facebook groups used to recruit participants and number of members at the time of recruitment.

**Additional file 2. **Interview guide translated from French.

## Data Availability

The datasets used and/or analyzed during the current study are available from the corresponding author on reasonable request.

## References

[1] Nicklaus S (2017). The Role of Dietary Experience in the Development of Eating Behavior during the First Years of Life. Ann Nutr Metab.

[2] Simeoni U, Bocquet A, Briend A, Chouraqui JP, Darmaun D, Dupont C (2016). L’origine précoce des maladies chroniques de l’adulte. Arch Pediatr.

[3] Blake-Lamb TL, Locks LM, Perkins ME, Woo Baidal JA, Cheng ER, Taveras EM (2016). Interventions for Childhood Obesity in the First 1,000 Days A Systematic Review. Am J Prev Med.

[4] Black RE, Victora CG, Walker SP, Bhutta ZA, Christian P, de Onis M (2013). Maternal and child undernutrition and overweight in low-income and middle-income countries. The Lancet.

[5] Singh AS, Mulder C, Twisk JW, van Mechelen W, Chinapaw MJ (2008). Tracking of childhood overweight into adulthood: a systematic review of the literature. Obesity reviews : an official journal of the International Association for the Study of Obesity.

[6] Abdullah A, Wolfe R, Stoelwinder JU, de Courten M, Stevenson C, Walls HL (2011). The number of years lived with obesity and the risk of all-cause and cause-specific mortality. Int J Epidemiol.

[7] Weihrauch-Blüher S, Schwarz P, Klusmann J-H (2019). Childhood obesity: increased risk for cardiometabolic disease and cancer in adulthood. Metabolism.

[8] Umer A, Kelley GA, Cottrell LE, Giacobbi P, Innes KE, Lilly CL (2017). Childhood obesity and adult cardiovascular disease risk factors: a systematic review with meta-analysis. BMC Public Health.

[9] Lioret S, Touvier M, Lafay L, Volatier J-L, Maire B (2008). Dietary and Physical Activity Patterns in French Children Are Related to Overweight and Socioeconomic Status. J Nutr.

[10] Lobstein T, Baur L, Uauy R (2004). Obesity in children and young people: a crisis in public health. Obesity reviews : an official journal of the International Association for the Study of Obesity.

[11] Bronfenbrenner U. The ecology of human development: Experiments in nature and design.: Cambridge, MA: Harvard University Press.; 1979.

[12] Wood AC, Blissett JM, Brunstrom JM, Carnell S, Faith MS, Fisher JO (2020). Caregiver Influences on Eating Behaviors in Young Children: A Scientific Statement From the American Heart Association. J Am Heart Assoc.

[13] Hetherington MM (2017). Understanding infant eating behaviour – Lessons learned from observation. Physiol Behav.

[14] Monnery-Patris S, Rigal N, Peteuil A, Chabanet C, Issanchou S (2019). Development of a new questionnaire to assess the links between children's self-regulation of eating and related parental feeding practices. Appetite.

[15] Brugaillères P, Chabanet C, Issanchou S, Schwartz C (2019). Caloric compensation ability around the age of 1 year: Interplay with the caregiver-infant mealtime interaction and infant appetitive traits. Appetite.

[16] McNally J, Hugh-Jones S, Caton S, Vereijken C, Weenen H, Hetherington M (2016). Communicating hunger and satiation in the first 2 years of life: a systematic review. Matern Child Nutr.

[17] DiSantis KI, Hodges EA, Johnson SL, Fisher JO (2011). The role of responsive feeding in overweight during infancy and toddlerhood: a systematic review. Int J Obes.

[18] Brugaillères P, Issanchou S, Nicklaus S, Chabanet C, Schwartz C (2019). Caloric compensation in infants: developmental changes around the age of 1 year and associations with anthropometric measurements up to 2 years. Am J Clin Nutr.

[19] Bell LK, Gardner C, Tian EJ, Cochet-Broch MO, Poelman AAM, Cox DN (2021). Supporting strategies for enhancing vegetable liking in the early years of life: an umbrella review of systematic reviews. Am J Clin Nutr.

[20] Haire-Joshu D, Tabak R (2016). Preventing Obesity Across Generations: Evidence for Early Life Intervention. Annu Rev Public Health.

[21] Smith JD, Fu E, Kobayashi MA (2020). Prevention and Management of Childhood Obesity and Its Psychological and Health Comorbidities. Annu Rev Clin Psychol.

[22] Schwartz C, Scholtens PA, Lalanne A, Weenen H, Nicklaus S (2011). Development of healthy eating habits early in life. Review of recent evidence and selected guidelines. Appetite.

[23] Fewtrell M, Bronsky J, Campoy C, Domellöf M, Embleton N, Fidler Mis N (2017). Complementary Feeding: A Position Paper by the European Society for Paediatric Gastroenterology, Hepatology, and Nutrition (ESPGHAN) Committee on Nutrition. J Pediatr Gastroenterol Nutr.

[24] Norton JL, Raciti MM (2017). Co-creating healthful eating behaviors with very young children: The impact of information overload on primary caregivers. Health Mark Q.

[25] Chouraqui J-P, Tavoularis G, Emery Y, Francou A, Hébel P, Bocquet M (2017). The French national survey on food consumption of children under 3 years of age – Nutri-Bébé 2013: design, methodology, population sampling and feeding practices. Public Health Nutr.

[26] Gildea A, Sloan S, Stewart M (2009). Sources of feeding advice in the first year of life: who do parents value?. Community practitioner : the journal of the Community Practitioners' & Health Visitors' Association.

[27] De Rosso S, Schwartz C, Ducrot P, Nicklaus S. The Perceptions and Needs of French Parents and Pediatricians Concerning Information on Complementary Feeding. Nutrients. 2021;13(7):2142. 10.3390/nu13072142. 10.3390/nu13072142 PMC8308433 34206652

[28] Nicklaus S, Remy E (2013). Early Origins of Overeating: Tracking Between Early Food Habits and Later Eating Patterns. Curr Obes Rep.

[29] ANSES. AVIS de l'Anses relatif à l'actualisation des repères alimentaires du PNNS - Jeunes enfants (0–3 ans). In: Agence nationale de sécurité sanitaire de l’alimentation, de l’environnement et du travail, editor. 2019. https://www.anses.fr/fr/content/avis-de-lanses-relatif-%C3%A0-lactualisation-des-rep%C3%A8res-alimentaires-du-pnns-jeunes-enfants-0-3. Accessed 20 Jul 2020.

[30] HCSP. AVIS relatif à la révision des repères alimentaires pour les enfants âgés de 0–36 mois et de 3–17 ans. 2020. https://www.hcsp.fr/explore.cgi/avisrapportsdomaine?clefr=924. Accessed 20 Nov 2020.

[31] De Rosso S, Nicklaus S, Ducrot P, Schwartz C. Information seeking of French parents regarding infant and young child feeding: practices, needs and determinants. Public Health Nutrition. 2022;25(4):879-92. 10.1017/S1368980021003086 PMC9991613 34321131

[32] Service-public.fr. Suivi médical de l'enfant : examens médicaux obligatoires 2020 [Available from: https://www.service-public.fr/particuliers/vosdroits/F967.

[33] Bocquet A, Vidailhet M. Nutri-Bébé 2013 Study Part 2. How do French mothers feed their young children? Archives de pediatrie : organe officiel de la Societe francaise de pediatrie. 2015;22(10 Suppl 1):10s7-s9. 10.1016/S0929-693X(15)30741-7 26474671

[34] INEPS. La santé vient en mangeant et en bougeant. Le guide nutrition des enfants et ados pour tous les parents.2004.

[35] Thomas DR (2006). A General Inductive Approach for Analyzing Qualitative Evaluation Data. Am J Eval.

[36] Draper A, Swift JA (2011). Qualitative research in nutrition and dietetics: data collection issues. J Hum Nutr Diet.

[37] Whitehead LC (2007). Methodological and ethical issues in Internet-mediated research in the field of health: An integrated review of the literature. Soc Sci Med.

[38] Tong A, Sainsbury P, Craig J (2007). Consolidated criteria for reporting qualitative research (COREQ): a 32-item checklist for interviews and focus groups. Int J Qual Health Care.

[39] INSEE. Toujours plus d’habitants dans les unités urbaines [Available from: https://www.insee.fr/fr/statistiques/4806684.

[40] INSEE. Taux de pauvreté selon l'âge du référent fiscal en 2018. Comparaisons régionales et départementales. [Available from: https://www.insee.fr/fr/statistiques/2012803.

[41] Toomey E, Flannery C, Matvienko-Sikar K, Olander EK, Hayes C, Heffernan T (2020). Exploring healthcare professionals’ views of the acceptability of delivering interventions to promote healthy infant feeding practices within primary care: a qualitative interview study. Public Health Nutr.

[42] MacMillan Uribe AL, Woelky KR, Olson BH (2019). Exploring Family-Medicine Providers’ Perspectives on Group Care Visits for Maternal and Infant Nutrition Education. J Nutr Educ Behav.

[43] Sjunnestrand M, Nordin K, Eli K, Nowicka P, Ek A (2019). Planting a seed - child health care nurses’ perceptions of speaking to parents about overweight and obesity: a qualitative study within the STOP project. BMC Public Health.

[44] van der Maas JC, Corbee RJ, Kroese FM, de Ridder DTD, Vos RC, Nielen M (2020). Discussing overweight in children during a regular consultation in general practice: a qualitative study. BMC Fam Pract.

[45] Braun V, Clarke V (2006). Using thematic analysis in psychology. Qual Res Psychol.

[46] Régnier F, Masullo A. Une affaire de goût ? Réception et mise en pratique des recommandations nutritionnelles. 2008.

[47] Dhuot R. La genèse précoce des différences sociales dans les habitudes alimentaires 2018.

[48] Tanaka T, Gjonça E, Gulliford MC (2012). Income, wealth and risk of diabetes among older adults: cohort study using the English longitudinal study of ageing. Eur J Pub Health.

[49] Bonaccio M, Bonanni AE, Di Castelnuovo A, De Lucia F, Donati MB, de Gaetano G (2012). Low income is associated with poor adherence to a Mediterranean diet and a higher prevalence of obesity: cross-sectional results from the Moli-sani study. BMJ Open.

[50] Barker D, Barker M, Fleming T, Lampl M (2013). Developmental biology: Support mothers to secure future public health. Nature.

[51] Cavalli B, De Lauzon-Guillain B, Turck D, Beghin L, Bonhoure S, Deplanque D (2017). Challenges encountered for implementing a public health intervention: The ECAIL study Difficultés rencontrées pour la réalisation d’une recherche interventionnelle en santé publique : l’étude ECAIL. Cahiers de Nutrition et de Diététique.

[52] Chouraqui J-P, Delmas B, Le Bris M, Bellaiche M, Jung C, Hanh T. Physicians advice, parental practice and adherence to doctor's advice: an original survey on infant feeding. BMC Pediatr. 2019;19(1):313-. 10.1186/s12887-019-1697-y PMC6724317 31484507

[53] Moore AP, Milligan P, Goff LM (2014). An online survey of knowledge of the weaning guidelines, advice from health visitors and other factors that influence weaning timing in UK mothers. Matern Child Nutr.

[54] Tarrant RC, Younger KM, Sheridan-Pereira M, White MJ, Kearney JM (2010). Factors associated with weaning practices in term infants: a prospective observational study in Ireland. Br J Nutr.

[55] Banti T, Carsin A, Chabrol B, Reynaud R, Fabre A. [Infant food diversification. Assessment of practices in relation to French recommendations in pediatricians and pediatric residents in southern France]. Archives de pediatrie : organe officiel de la Societe francaise de pediatrie. 2016;23(10):1018–27. 10.1016/j.arcped.2016.07.007 27642151

[56] Régnier F. Social Appropriation of “Diet and Health” Information: From Public Health Campaigns to Digital Tools. Food and Health2019. p. 217–38.

