# Supplementary material for: Counseling parents about child feeding: a qualitative evaluation of French doctors and health/childcare professionals’ experiences and perception of a brochure containing new recommendations
Source: BMC Public Health. 2022 Dec 8;22:2303. doi: 10.1186/s12889-022-14778-2 (PMC9733000; doi:10.1186/s12889-022-14778-2)
Supplement: Supplementary file 1 — Additional file 1. Facebook groups used to recruit participants and number of members at the time of recruitment. [file 12889_2022_14778_MOESM1_ESM.pdf]

**De Rosso S, Riera-Navarro C, Ducrot P, Schwartz C, Nicklaus S. Counseling parents about child feeding: a qualitative evaluation of French doctors and health/childcare professionals' experiences and perception of a brochure containing new recommendations.**

**Additional File 1.** Facebook groups used to recruit participants and number of members at the time of recruitment.

| Facebook group | Number of members |
|---|---|
| Echanges de docs entre professionnels de la petite enfance | 25289 |
| Objectif Thése Médecins – Diffuse ton questionnaire auprès de médecins | 859 |
| Les Médecins de Facebook | 20308 |
| Association des Jeunes Médecins Généralistes de Bourgogne - AJMGB | 995 |
| Propositions de remplacements en Rhône-Alpes pour jeunes généralistes | 2527 |
| Le cercle des pédiatres | 1456 |
| Remplacement médecine générale paris/idf | 2125 |
| Avis medical entre médecins : specialists, généralistes et même internes | 4102 |
