# Supplementary material for: Counseling parents about child feeding: a qualitative evaluation of French doctors and health/childcare professionals’ experiences and perception of a brochure containing new recommendations
Source: BMC Public Health. 2022 Dec 8;22:2303. doi: 10.1186/s12889-022-14778-2 (PMC9733000; doi:10.1186/s12889-022-14778-2)
Supplement: Supplementary file 2 — Additional file 2. Interview guide translated from French. [file 12889_2022_14778_MOESM2_ESM.pdf]

De Rosso S, Riera-Navarro C, Ducrot P, Schwartz C, Nicklaus S. Counseling parents about child feeding: a qualitative evaluation of French doctors and health/childcare professionals' experiences and perception of a brochure containing new recommendations

Additional File 2. Interview guide translated from French.

**SEMI-STRUCTURE INTERVIEW GUIDE**

> **Note:** The interview guide presented in the following pages is intended as a checklist of points to be addressed during the interview. It is by no means a questionnaire that would be repeated line by line. The level of language, the questions, and the time allotted to each part should be adapted to the interviewee.
>
> The order of the follow-up questions can also be modified according to the respondent's answers.

1. Introduction researcher + study/ consent form/ questions.

Hello, my name is ....., I work at the Centre des Sciences du Goût et de l'Alimentation in Dijon. First of all, thank you for agreeing to participate in this video call. I am conducting interviews to learn more about your needs in terms of communication related to food for 0-3 year olds. I propose that we discuss your communication practices with parents of children aged 0-3 years. I would like to specify that everything you tell me will remain between us, our exchanges will be used for my study but will remain anonymous. If you agree, I will record this discussion to make sure that I do not distort your answers in the transcription. By agreeing to continue the interview, you are agreeing to have your voice recorded and kept in our archives until the end of 2021. Okay, are you ready?

Note: Part 2 of the guide should not exceed 15 minutes of interview time. The questions will be selected based on the respondent's answers. It is not necessary to ask all the questions in this section.

2. Questions on perceptions and practices related to the needs of doctors and health/childcare professionals in terms of communication in relation to the feeding of 0-3 year olds

1) Can you tell me about your experience in general, in terms of your communication with parents on the subject of feeding children aged 0-3 years?

Note: For childcare personnel, specify that we are going to discuss their communication with parents, i.e. the exchanges they have with parents, the information and advice they give them (and not the practices they implement at the daycare center to feed the children).

If not enough information: For doctors reiterate with: *Think back to the last time(s) you had a consultation with a parent during which you discussed feeding 0-3 year olds. Can you tell me about that consultation?*

For health/childcare professionals in the field of early childhood, repeat with: *How have you responded to the needs of parents who ask for information about feeding children from 0 to 3 years old?*

2) Do you give information and advice to parents on nutrition spontaneously and systematically (if so, explain how, at what key ages) or do you bring up this subject rather in response to

parents' questions? (if so, what questions from parents come up)

3) Do you have the time to adequately answer parents' questions about feeding?

4) Do you see more patients/families with high or low socio-economic levels, or both? Do you see migrants? Do you encounter cultural differences? Language barriers? How do you deal with this?

5) How do you communicate information or advice to parents about feeding 0-3 year olds? Do you use any materials in addition to oral communication? If so, what kind of materials?

(Brochures? Websites? etc.). Does the type of support depend on the families?

If they say they have their own materials/booklet that they give to parents, ask where they got the information to build the document

6) In your experience, what are the barriers (obstacles/problems) you encounter when you have to inform or advise parents about feeding their child between 0 and 3 years old?

Do you feel that your advice is understood and accepted by parents? Especially by parents from lower socio-economic levels for example?

What could be done to facilitate acceptance of the advice and improve your communication as... (pediatricians / general practitioners / health/childcare professionals) with parents on this topic of feeding 0-3 year olds?

7) From your experience and discussions with parents,

What do you think of the information available to parents? Where do parents get information about feeding 0-3 year olds?
What do you think could be improved?

3. Questions about whether communication patterns with parents may have changed after the Covid-19 health crisis.

Announce this new topic to the participant. *In the past year there have been many changes due to restrictions following the emergence of covid, etc.*

<u>For early childhood pros</u>:

- Have your professional practices been impacted by Covid-19? What has changed?

- Has your communication with parents about feeding children aged 0 to 3 years been impacted? What has changed?

- Have you noticed, with the health crisis, new concerns from parents about their children's dietary needs (perhaps they thought they needed to change something)

- In this past year, have parents turned to you more for advice? IF YES: Why do you think that is?

<u>For doctors (pediatricians and general practitioners):</u>

- Have your professional practices been impacted by Covid-19? What has changed?

- Has your communication with parents about feeding children aged 0-3 years been impacted? What has changed?

- Have you increased the number of teleconsultations (consultations by phone or video calls)? If so, how did you feel about it?

- During the teleconsultation, were you able to discuss their child's feeding with the parents?

- Were there any differences from face-to-face consultations? For example: Do you discuss the same topics?

- Do you feel that communication via teleconsultation is as effective as in-person consultations?

4. Questions to assess the level of understanding and consent of a parent-targeted brochure (Professionals will receive the brochure in pdf by email at least two weeks before the interview)

<u>Overall spontaneous perception of the brochure</u>

- Did you read the brochure we sent? Overall, what did you think of the manuscript of this future brochure?

LET THE PARTICIPANTS EXPRESS THEMSELVES FREELY AT FIRST, THEN ASK THEM AGAIN:

<u>Comprehension and memorization</u>

- What elements of this brochure particularly impressed you? Why or why not?

- Were there things that surprised you in this brochure? Which ones? Why or why not?

- Did you notice anything new in the recommendations? Which ones? What do you think of this/these information?

ASK AGAIN:

- Among the new features/new recommendations, there is for example the notion of: Do not start food diversification after 6 months (except in special cases of course...).

What do you think about this information? Do you think that the explanation to understand this notion is clear? Do you think that this can be clear for the parents or is it better to give more information about it? (See if allergy prevention has been retained)

- Another new concept is to introduce all food groups between 4 and 6 months. What do you think of this information? Do you need information to understand why?

- You may have seen that there is also talk of introducing foods like pulses, potatoes, whole grains. (What do you think about this information? Do you need more information to understand why?

- Another point I wanted to share with you is the fact that we suggest alternating growing-up milk with whole cow's milk UHT. What do you think about this information? Do you need information to understand why?

- And in terms of foods that are not recommended, what do you think of the information we give? In particular for sweetened products, and sweetened drinks including fruit juices? For ultra-processed products? Soy products and vegetable drinks? Certain fish? Do you need information to understand why?

- If not, is there anything in this brochure that you found difficult to understand or that did not seem clear? Which ones?

<u>Accreditation and Membership</u>

- What did you like about this brochure? And what did you like or dislike? Why or why not?

MORE SPECIFIC QUESTIONS :

- What do you think of the vocabulary used? ASK AGAIN: *Are the messages clear and easy to understand for parents?*

- What do you think of the tone used in the brochure?

- What do you think of the information given? ASK AGAIN*: Are there any important things missing/that you feel are important to communicate to parents?*

- *What do you think of the order in which the different topics are presented?*

- *What would you do to improve this brochure?*

Usefulness of the brochure for parents and professionals

- *Are these messages useful? Why or why not?*

- *Do you feel that parents can learn things? What can they learn?*

- *What do you think this brochure makes parents want to do? Will they change things about their children's nutrition on a daily basis? (*YOU MAY ASK AGAIN: CHANGE DAILY FEEDING, DISCUSS WITH SPOUSE, HEALTH PROFESSIONAL, FRIENDS, RELATIVES...*)*

For health/childcare professionals:

- *Finally, do you think it would be helpful for you to have this brochure to give to parents? Why or why not?*

For doctors

- *Do you think this type of brochure is useful for you during consultations with parents? Why or why not?*

- *Finally, there are some tips that are difficult to explain in a brochure and that we thought we would try to pass on to parents through doctors, for example how to introduce certain allergens such as nuts or for example chocolate to avoid.*
  - *o Would you agree to relay this type of advice to parents*
  - *o Do you feel that this is your role? Who else could relay it? How else could we do it?*
  - *o What would you need to make it easy for you to give this information?*

For all:

- *If they say they have created their own materials to give to parents, ask them if they would replace those materials with the brochure.*

- *Will the booklet make them change any of the practices/advice they give to parents*

5. Additional comments/thoughts from the participant

- *Is there anything I haven't mentioned that you would like to add?*

6. Conclusion of the interview + thanks and compensation + explanation of what to do next

- *If you have nothing further to add, I thank you very much for your time and for answering my questions.*

- *To compensate you for your time, I would like to offer you Amazon vouchers for a total amount of 40€.*

- *Do you still have questions about the study in general?*
